# Advanced Amperometric Microsensors for the Electrochemical Quantification of Quercetin in *Ginkgo biloba* Essential Oil from Regenerative Farming Practices

**DOI:** 10.3390/metabo15010006

**Published:** 2024-12-31

**Authors:** Elena Oancea, Ioana Adina Tula, Gabriela Stanciu, Raluca-Ioana Ștefan-van Staden, Jacobus (Koos) Frederick van Staden, Magdalena Mititelu

**Affiliations:** 1 Laboratory of Electrochemistry and Condensed Matter, National Institute of Research for Electrochemistry and Condensed Matter, 202, Splaiul Independentei Street, 060021 Bucharest, Romania; elena@carelessbeauty.ro (E.O.); adina@carelessbeauty.ro (I.A.T.); koosvanstaden@patlab.ro (J.F.v.S.); 2 SC. Dan-Elis. SRL, Cosmetic Product Manufacturing, 907285 Topraisar, Romania; 3 Department of Chemistry and Chemical Engineering, Ovidius University of Constanta, 900527 Constanta, Romania; 4 Laboratory of Electrochemistry and PATLAB, National University of Science and Technology Politehnica of Bucharest, 060042 Bucharest, Romania; 5 Department of Clinical Laboratory and Food Safety, Faculty of Pharmacy, “Carol Davila” University of Medicine and Pharmacy, 020956 Bucharest, Romania; magdalena.mititelu@umfcd.ro

**Keywords:** carbon nanopowder, chitosan, differential pulse voltammetry, essential oils, graphite, tetraphenyl-porphine cobalt (II), quercetin

## Abstract

In this study, we present a novel approach using amperometric microsensors to detect quercetin in cosmetic formulations and track its metabolic behavior after topical application. This method offers a sensitive, real-time alternative to conventional techniques, enabling the detection of quercetin’s bioavailability, its transformation into active metabolites, and its potential therapeutic effects when applied to the skin. Quercetin (Q) is a bioactive flavonoid known for its potent antioxidant properties, naturally present in numerous plants, particularly those with applications in cosmetic formulations. In response to the growing interest in developing novel plant-based dermo-cosmetic solutions, this study investigates the electrochemical detection of quercetin, a ketone-type flavonoid, extracted from Gingko biloba essential oil. Three newly designed amperometric microsensors were developed to assess their efficacy in detecting quercetin in botanical samples. The sensor configurations utilized two forms of carbon material as a foundation: graphite (G) and carbon nanoparticles (CNs). These base materials were modified with paraffin oil, chitosan (CHIT), and cobalt(II) tetraphenylporphyrin (Co(II)TPP) to enhance sensitivity. Differential pulse voltammetry (DPV) served as the analytical method for this investigation. Among the sensors, the CHIT/G–CN microsensor exhibited the highest sensitivity, with a detection limit of 1.22 × 10^−7^ mol L^−1^, followed by the G–CN (5.64 × 10^−8^ mol L^−1^) and Co(II)TPP/G–CN (9.80 × 10^−8^ mol L^−1^) microsensors. The minimum detectable concentration was observed with the G–CN and CoP/G–CN microsensors, achieving a threshold as low as 0.0001 μmol L^−1^. Recovery rates and relative standard deviation (RSD) values averaged 97.4% ± 0.43, underscoring the sensors’ reliability for quercetin detection in botanical matrices.

## 1. Introduction

The growing demand for natural ingredients in cosmetic formulations has led to the increased use of plant-derived antioxidants, such as quercetin, a flavonoid with known antioxidant and anti-inflammatory properties. Given the rising interest in cosmeceuticals, there is a pressing need for advanced, accurate methods to detect these compounds in cosmetic matrices and track their absorption, bioavailability, and potential metabolic transformations in the skin [1,2].

Flavonoids, found in a variety of plant-based foods, are well-known for their potent antioxidant activity. Present in abundant quantities in fruits and vegetables, like leafy greens, berries, and citrus fruits, flavonoids play a critical role in neutralizing free radicals and preventing damage to vital cellular structures, such as DNA, lipids, and proteins. Additionally, phenolic acids contribute significantly to antioxidant defense by acting as reducing agents, quenching reactive oxygen species (ROS), and chelating metal ions, further enhancing their protective properties [3,4,5,6].

Among these, quercetin has been extensively studied for its broad pharmacological effects. Known for its antioxidant properties, quercetin helps protect skin cells from oxidative damage caused by external factors, such as UV radiation and pollution. Its rapid absorption into the skin allows for immediate protection, making it a valuable ingredient in topical formulations aimed at safeguarding skin health [7,8].

Beyond its antioxidant action, quercetin exhibits a range of therapeutic benefits, including anti-inflammatory effects that can calm irritated skin and reduce redness. It also shows promise in regulating blood pressure, improving vascular function, and even addressing obesity. These diverse biological activities highlight quercetin’s potential not only as a cosmetic ingredient, but also as a therapeutic agent in managing oxidative stress-related chronic conditions [3,9].

However, integrating flavonoids like quercetin into cosmetic products poses challenges related to their stability, bioavailability, and skin penetration. Flavonoids are prone to degradation when exposed to light, heat, or air, potentially reducing their effectiveness unless properly stabilized. To address these concerns, various delivery systems—such as liposomal encapsulation and nanoparticles—have been explored to enhance the bioavailability and stability of flavonoids, ensuring their deeper penetration into the skin for more pronounced effects [10,11].

In parallel, advancements in electrochemical detection methods have enabled precise measurement of quercetin and other flavonoids in plant extracts, offering insights into their concentration and bioactivity. These developments are essential for optimizing extraction processes and improving formulation techniques, ensuring that cosmeceuticals contain effective levels of bioactive compounds [12,13,14].

While numerous techniques, such as high-performance liquid chromatography (HPLC) and spectrophotometry, have been used to detect quercetin in botanical extracts, these methods primarily focus on its precursors without addressing how quercetin or its metabolites are processed after topical application in cosmetics. This gap in the knowledge limits our understanding of how quercetin interacts with the skin and potentially enters the systemic circulation, where its metabolites could exert additional therapeutic effects [15].

In this study, we present a novel approach using amperometric microsensors to detect quercetin in cosmetic formulations and track its metabolic behavior after topical application. This method offers a sensitive, real-time alternative to conventional techniques, enabling the detection of quercetin’s bioavailability, its transformation into active metabolites, and its potential therapeutic effects when applied to the skin.

This study aims to extend the application of electrochemical detection to quercetin, a flavonoid with significant cosmetic potential, extracted from *Ginkgo biloba* essential oil. Three novel amperometric microsensors were designed, based on graphite (G) and carbon nanoparticles (CNs), with configurations including both unmodified and modified versions using chitosan (CHIT) and cobalt(II) tetraphenylporphyrin (Co(II)TPP). Differential pulse voltammetry (DPV) was chosen as the primary electrochemical method for detecting quercetin, building upon the protocol by Van Staden et al. (2014) [16], initially developed for serotonin detection in biological fluids and adapted here for quercetin in a botanical matrix. The selection of chitosan and Co(II)TPP was based on their proven stability and effectiveness in acidic environments, crucial for the analysis of quercetin in essential oils.

By optimizing these electrochemical detection techniques, this study aims to ensure the precise measurement of quercetin and facilitate its incorporation into cosmeceutical products, further enhancing the analysis of plant-based bioactive compounds.

Quercetin, a widely studied flavonoid found in various fruits, vegetables, and medicinal plants, has garnered significant attention for its antioxidant and therapeutic properties. Known for its ability to scavenge free radicals, quercetin helps protect the body from oxidative stress, a key factor in aging and the development of chronic diseases like cardiovascular disorders and cancer. While its biological effects have been extensively explored in previous studies, the focus of this research lies in the development of electrochemical detection methods to quantify quercetin in complex matrices such as essential oils [8,17].

The main challenge in detecting quercetin within such matrices is the compound’s complex chemical nature, which can complicate its extraction and analysis. As quercetin is sensitive to environmental factors like light and heat, its incorporation into essential oils requires accurate and reliable methods to ensure the compound remains stable and detectable. Furthermore, understanding the best techniques for quantifying quercetin, especially in botanical formulations, is critical for optimizing its use in therapeutic and cosmetic applications. This study addresses these challenges by developing novel amperometric microsensors to enhance the precision and sensitivity of quercetin detection, providing a valuable tool for future research and product development in the field of cosmeceuticals and natural health products.

The accurate quantification of quercetin in cosmetic products is essential for ensuring consistent product quality and efficacy. The ability to measure trace amounts of quercetin and its metabolites is critical for product formulation, quality control, and ensuring that these compounds exert the desired antioxidant and anti-inflammatory effects on the skin [17].

The goal of this study is to develop a highly sensitive electrochemical approach for detecting quercetin in *Ginkgo biloba* essential oil, providing a robust tool for quantifying both the parent compound and its metabolites in cosmeceutical formulations. Through this method, we aim to advance the field of metabolite science, shedding light on quercetin’s absorption, metabolism, and bioactivity in cosmetic applications.

*Ginkgo biloba*, one of the oldest living tree species, is native to China but has also been cultivated in other parts of Asia, Europe, and North America. It is commonly known as the “maidenhair tree” due to the distinctive fan-shaped leaves. The plant has been revered in traditional medicine for centuries, with a long history of use in both Eastern and Western herbal practices [18].

Various parts of the *Ginkgo biloba* tree, including the leaves, seeds, and bark, have been used in traditional medicine, though it is primarily the leaves that are exploited for their medicinal properties. Ginkgo leaf extracts are rich in bioactive compounds, notably flavonoids, terpenoids, and ginkgolides, which are thought to contribute to the plant’s therapeutic effects. These extracts are commonly used in modern medicine, particularly in the treatment of cognitive disorders, circulatory problems, and as an antioxidant agent. They have been extensively researched for their potential neuroprotective, anti-inflammatory, and cardioprotective benefits [19,20].

In the cosmetic industry, *Ginkgo biloba* extracts are utilized for their skin-protective properties. The antioxidants, particularly flavonoids like quercetin, are valued for their ability to neutralize free radicals, which helps to reduce oxidative stress and protect the skin from environmental damage, such as UV radiation and pollution. As such, *Ginkgo biloba* extract is frequently incorporated into skincare products aimed at anti-aging, skin hydration, and improving skin elasticity [21].

In countries where *Ginkgo biloba* grows naturally, such as China and parts of Japan, the plant is also used in folk medicine. Traditional uses of the plant include treatment for respiratory issues, as a general tonic, and for its purported benefits in improving memory and mental function [22]. The growing interest in *Ginkgo biloba*’s secondary metabolites, particularly in the context of their antioxidant properties, has further expanded its applications in both medicine and cosmetics worldwide [23].

Adding a discussion of the plant’s widespread use in traditional and modern medicine will provide valuable context for understanding its relevance in the study of secondary metabolites, particularly quercetin, and its role in health and skincare products.

## 2. Materials and Methods

### 2.1. Reagents, Materials, and Solutions

This study involved the development of three innovative modified amperometric microsensors tailored for the electrochemical detection of quercetin (Q) in botanical samples, specifically using extracts from *Gingko biloba* essential oil. The study design incorporated materials, chemicals, and solutions adapted from the methodology established by Van Staden et al. (2014) [16].

The following reagents were procured from Sigma Aldrich: quercetin (Q), graphite powder (1–2 mm, synthetic) for the sensor base material, carbon nanoparticles (CNs) with a particle size of less than 50 nm (characterized by transmission electron microscopy—TEM), low-molecular-weight chitosan (CHIT), tetraphenylporphyrin cobalt(II) (Co(II)TPP), as well as KCl, NaCl, NaNO_3_, NaH_2_PO_4_·H_2_O, Na_2_HPO_4_·7H_2_O, ethanol, and dimethyl sulfoxide. Paraffin oil was sourced from Fluka and served as the binder in the paste matrix.

Graphite (G) was selected for its high electrical conductivity, while chitosan (CHIT) and cobalt(II) tetraphenylporphyrin (Co(II)TPP) were incorporated as functional modifiers due to their notable stability, compatibility with acidic environments, and extensive use in pharmaceutical and biomedical applications.

All solutions were prepared using deionized water obtained through a Direct-Q 3 Water Purification system (Millipore Corporation, France). Phosphate buffer solutions (0.2 M, pH 1 to 10) were prepared based on the established protocols, using specific molar ratios of NaH_2_PO_4_ and Na_2_HPO_4_. pH adjustments were made by adding small quantities of 0.1 M NaOH or HCl as needed.

The *Gingko biloba* essential oil was supplied by Careless Beauty Romania, a cosmetics manufacturer. Raw materials were harvested from a private regenerative farm located in the Topraisar Village, Constanța County, Romania. The harvested material was thoroughly purified and subjected to a hydrodistillation process to obtain the essential oil in its fresh state.

A stock solution of quercetin (Q) at a concentration of 1000 µmol L^−1^ (10^−3^ mol L^−1^) was prepared by dissolving 10 mL of the compound. Serial dilutions were then conducted from this stock solution to achieve a range of concentrations between 0.0001 and 1000 µmol L^−1^ (10^−10^ to 10^−3^ mol L^−1^). These solutions were subsequently adjusted to different pH values using a phosphate buffer and included 0.1 mol L^−1^ concentrations of KCl, NaCl, and NaNO_3_ as electrolytes.

### 2.2. Design of the Unmodified and Modified Amperometric Microsensors

**Figure 1:**
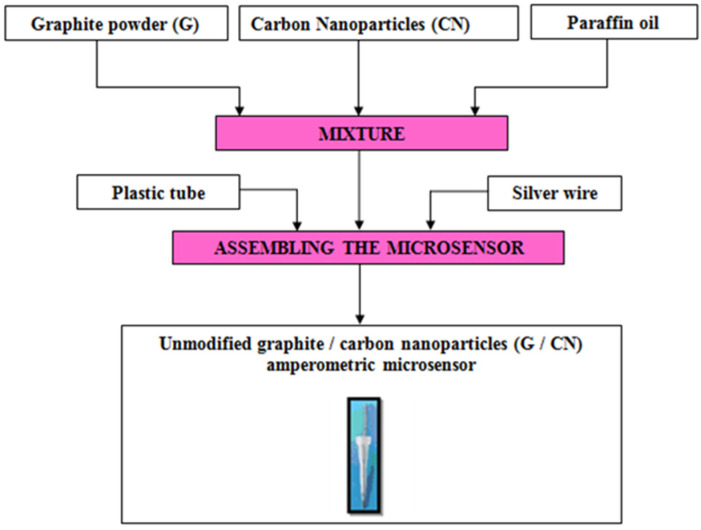
Protocol for the design of the unmodified graphite/carbon nanoparticle (G/CN) amperometric microsensor.

**Figure 2:**
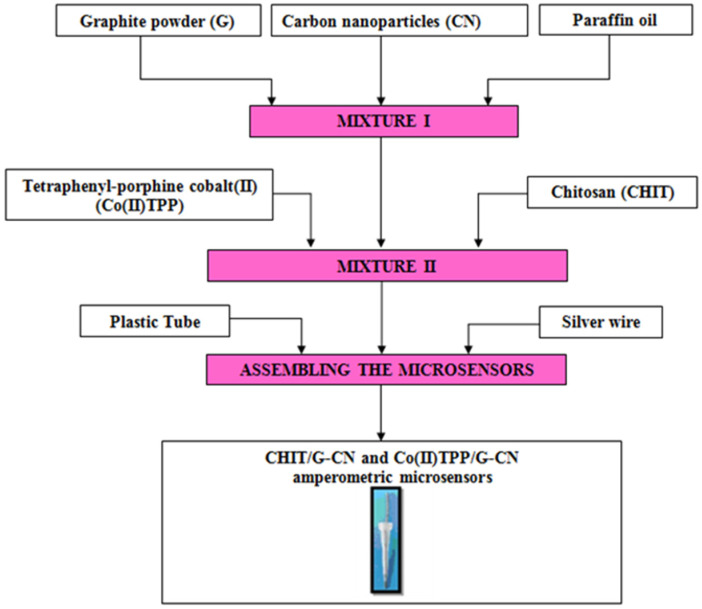
Protocol for the design of the graphite (G)/carbon nanoparticle (CN) modified with chitosan (CHIT) (CHIT/G-CN) and tetraphenyl-porphine cobalt(II) (Co(II)TPP) (Co(II)TPP/G-CN) amperometric microsensors.

The fabrication of both unmodified and modified amperometric microsensors followed a well-established protocol previously detailed in our earlier research [24,25,26,27]. Accordingly, the protocol to design the three amperometric microsensors based on the graphite (G) and carbon nanoparticle paste mixture is described in Figure 1 and Figure 2.

### 2.3. Instrumentation

For the amperometric measurements, we used an IVIUM COMPACTSTAT potentiostat connected to a three-electrode cell that was connected to a computer using the IVIUM software version 2.025. A silver/silver chloride electrode served as the reference electrode, and a platinum electrode was employed as the auxiliary electrode. The pH measurements were obtained by use of a CyberScan PCD 6500 Multiparameter meter (Eutech instruments, Singapore).

All of the differential pulse voltametric (DPV) measurements were performed at 25 °C employing a scan rate of 50mV s^−1^, a pulse height of 0.025 V, and a step time of 0.2 s.

**Figure 3:**
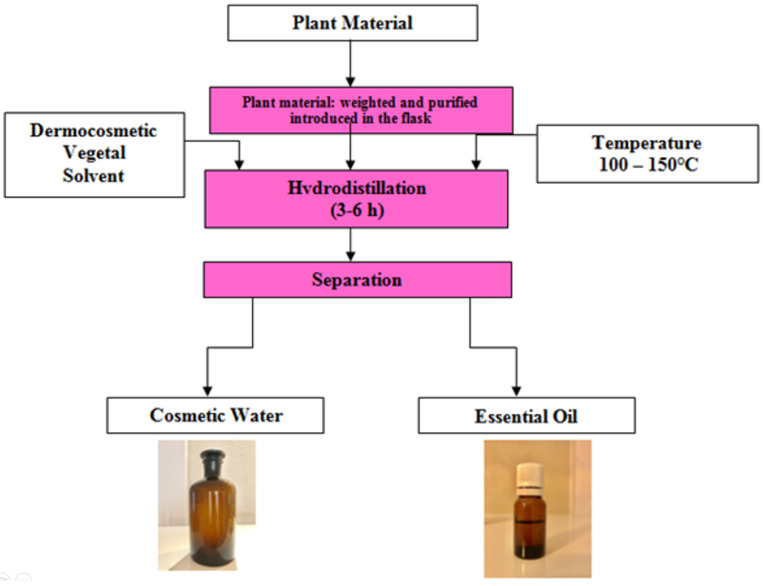
Neoclevenger hydrodistillation technologic process.

The *Gingko biloba* essential oil was obtained using a Neoclevenger hydrodistillation system adapted for sensitive plant extractions following the technologic process described in Figure 3. The pH and ORP (oxido-reduction potential) measurements of the plant-source essential oils were performed using a meter containing a combined electrode from Oakton, USA.

### 2.4. Sample Preparation

The *Ginkgo biloba* essential oil, sourced from Careless Beauty Romania, was prepared for analysis by buffering the samples with a phosphate solution at pH 3.00. This buffer included 0.1 mol L^−1^ NaCl, and an additional preparation at pH 3 containing 0.1 mol L^−1^ KCl. These conditions, along with the differential pulse voltammetry (DPV) parameters previously described, were utilized to quantify quercetin (Q) in the botanical sample.

Each sample was analyzed in triplicate to ensure precision and reliability. A total of 6 independent samples of *Ginkgo biloba* essential oil were tested, with each being processed and analyzed three times (*n* = 3). This resulted in 18 individual measurements for the validation of the method.

All determinations were performed in triplicate, and the results were expressed as mean ± SD (standard deviation). A statistical evaluation of clinical results was performed by the Student’s *t*-test and analysis of variance (ANOVA).

## 3. Results

### 3.1. Optimization of the Analytical Conditions

To optimize the analytical conditions, DPV anodic curves were obtained for solutions that contained 100 μmol L^−1^ (10^−4^ mol L^−1^) of quercetin (Q) using different supporting electrolytes in 0.2 mol L ^−1^ of phosphate buffer solutions with pH values between 1 and 10. Three supporting electrolytes were used to optimize this parameter: 0.1 mol L^−1^ KCl, 0.1 mol L^−1^ NaCl, and 0.1 mol L^−1^ NaNO_3_. The influence of the pH and supporting electrolyte on the peak height was evaluated for the amperometric microsensors based on carbon materials (e.g., graphite (G) and carbon nanoparticles (CNs) unmodified and modified with chitosan (CHIT) and tetraphenyl-porphine cobalt(II) (Co(II)TPP)).

**Figure 4:**
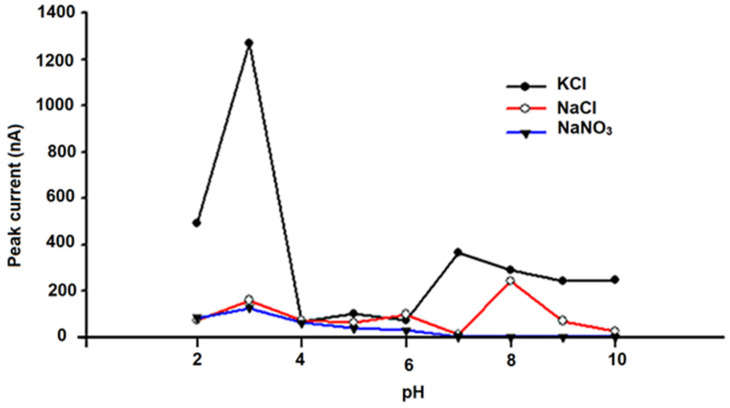
Performance of the unmodified graphite/carbon nanoparticle (G-CN) amperometric microsensor was evaluated using three different electrolytes across a range of pH levels in the 10^−4^ mol/L quercetin (Q) solution.

**Figure 5:**
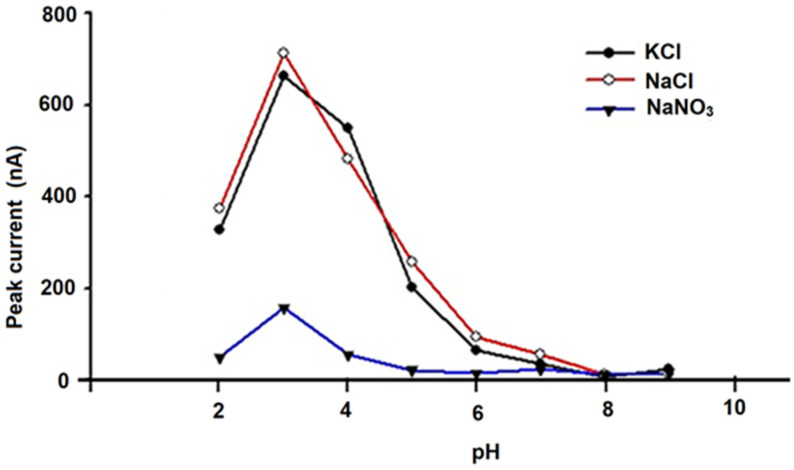
Performance of the chitosan-modified graphite/carbon nanoparticle (CHIT/G-CN) amperometric microsensor evaluated using three types of electrolytes at various pH values in the 10^−4^ mol/L quercetin (Q) solution.

**Figure 6:**
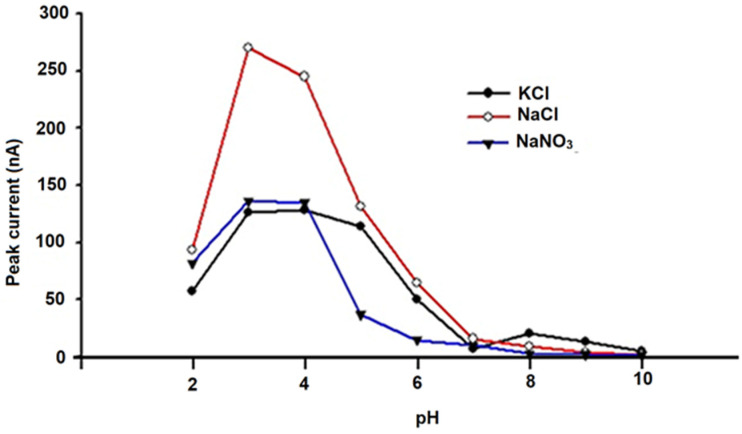
Performance of the tetraphenyl-porphine cobalt (III)-modified graphite/carbon nanoparticle (Co(II)TPP/G-CN) amperometric microsensor evaluated using three types of electrolytes at various pH values in the 10^−4^ mol/L quercetin (Q) solution.

The results shown in Figure 4, Figure 5 and Figure 6 demonstrate that optimum responses are observed at pH 3.00 and 0.1 mol L^−1^ NaCl for the unmodified G-CN microsensor, and also at pH 3.00 and 0.1 mol L^−1^ KCl for the modified (CHIT/G—CN) and (Co(II)TPP)/G-CN microsensors.

As a result, these conditions were chosen for further investigation, factoring in the naturally acidic pH of the Gingko biloba essential oil botanical sample included in this study.

### 3.2. Analytical Performance for the Differential Pulse Anodic Voltammetric Measurements of Quercetin (Q)

Differential pulse anodic voltammetry (DPV) measurements were conducted using three types of amperometric microsensors based on graphite (G) and carbon nanoparticles (CNs). These microsensors included an unmodified G-CN version, a chitosan-modified version (CHIT/G-CN), and a version modified with tetraphenylporphyrin cobalt(II) (Co(II)TPP/G-CN). These experiments aimed to verify the prior findings and further characterize the microsensors’ analytical performance.

**Figure 7:**
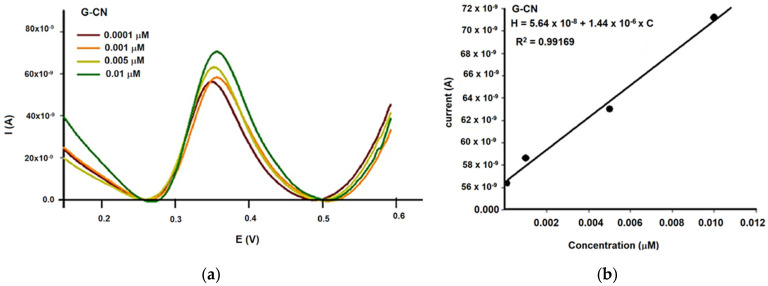

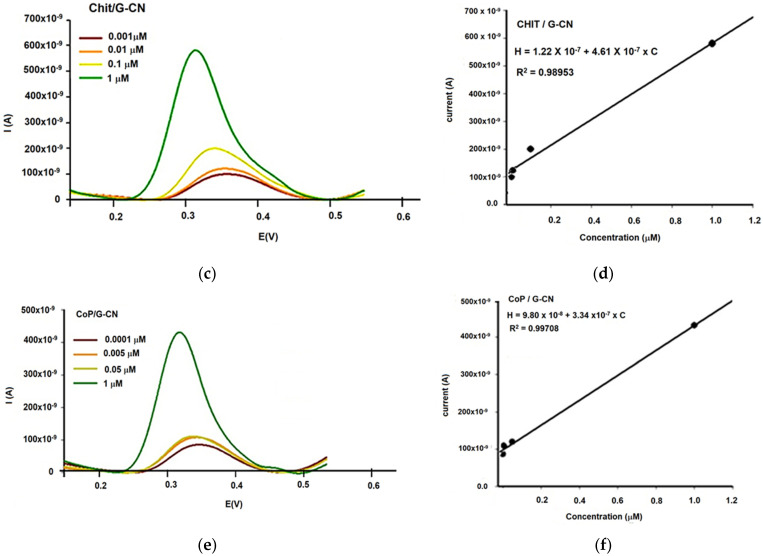
Representative differential pulse voltammograms (**a**) and calibration curves (**b**) for quercetin (Q) detection using the graphite/carbon nanoparticle (G/CN) amperometric microsensor; representative differential pulse voltammograms (**c**) and calibration curves (**d**) for quercetin (Q) detection using the graphite/carbon nanoparticle amperometric microsensor modified with chitosan (CHIT/G-CN); representative differential pulse voltammograms (**e**) and calibration curves (**f**) for quercetin (Q) detection, obtained using the graphite–carbon nanoparticles microsensor modified with tetra-phenyl-porphine cobalt(II) (Co(II)TPP/G-CN).

Figure 7 displays typical calibration peaks obtained with DPV for quercetin solutions in the range of 0.0001 to 1 mol/L, using each of the three sensor configurations: unmodified G-CN (Figure 7a,b), chitosan-modified G-CN (CHIT/G-CN, Figure 7c,d), and tetraphenylporphyrin cobalt(II)-modified G-CN (Co(II)TPP/G-CN, Figure 7e,f). The DPV measurements were performed at a scan rate of 50 mV s^−1^. The results demonstrate a direct proportionality between the DPV current response and quercetin concentration.

### 3.3. Response Characteristics of the Amperometric Microsensors

The influence on carbon materials matrices (e.g., graphite (G) and carbon nanoparticles (CNs), and modifiers such as chitosan (CHIT) and tetraphenyl-porphine cobalt(II) (Co(II)TPP)) was studied using three amperometric microsensors for the determination of quercetin (Q). For the reliability and stability, the working concentrations covered by the proposed microsensors for the assay of quercetin (Q) were between 0.0001 and 1 μmol L^−1^ as shown in Table 1.

The highest sensitivity was observed with the CHIT/G–CN amperometric microsensor, achieving a detection threshold of 1.22 × 10^−7^ mol L^−1^, followed by the G–CN (5.64 × 10^−8^ mol L^−1^) and Co(II)TPP/G–CN (9.80 × 10^−8^ mol L^−1^) microsensors. The detection limit was determined in accordance with Otto’s recommendations [28]. The lowest detection limit was identified for both G–CN (0.0001 µmol L^−1^) and CoP/G–CN (0.0001 µmol L^−1^) microsensors.

The amperometric microsensors demonstrated stable performance over three months of continuous use, with sensitivity values exhibiting a relative standard deviation (RSD) of less than 1%.

### 3.4. Method Validation

Three types of amperometric microsensors, based on graphite (G), carbon nanoparticles (CNs) (G–CN), and modified variants with chitosan (CHIT/G–CN) and tetraphenylporphyrin cobalt(II) (Co(II)TPP/G–CN), were evaluated for the electrochemical detection of quercetin (Q) in a botanical sample intended for cosmetic applications. Recovery rates are detailed in Table 2.

## 4. Discussion

The results of this study demonstrate that quercetin (Q) was successfully assayed in *Ginkgo biloba* essential oil, with recovery rates reaching up to 97.4%. Among the different microsensors tested, the graphite and carbon nanoparticle (G-CN) sensor yielded the highest recovery, followed by the chitosan (CHIT)/G-CN and cobalt(II) tetraphenylporphyrin (Co(II)TPP)/G-CN sensors.

In this research, three distinct amperometric microsensors were developed to evaluate the electrochemical characteristics of quercetin in Ginkgo biloba oil. The base sensors, composed of graphite (G) and carbon nanoparticles (CNs), provided a robust and conductive platform. Modifications with chitosan (CHIT) and Co(II)TPP enhanced their sensitivity and selectivity. Chitosan, a biopolymer derived from chitin, was chosen for its biocompatibility and capacity to improve electron transfer, making it an ideal material for biosensor applications. Similarly, Co(II)TPP, a metalloporphyrin, was selected to improve electrocatalytic activity, enabling the detection of quercetin at lower concentrations.

The differential pulse voltammetry (DPV) technique applied in this study was particularly effective for detecting quercetin due to its high resolution and sensitivity. DPV works by applying a series of potential pulses to the electrochemical cell, allowing for the detection of current changes as quercetin undergoes oxidation or reduction. This method is well-suited for detecting low concentrations of analytes, which is essential in cosmeceutical and pharmaceutical research, where the precise quantification of active compounds is critical. Previous studies have demonstrated DPV’s effectiveness in detecting flavonoids like quercetin, where sensitivity and selectivity are crucial for analyzing complex biological samples [29,30,31]. Similarly, other research has employed DPV for monitoring antioxidant levels in pharmaceutical and food matrices [32,33,34], highlighting the method’s potential for trace-level analysis.

In our study, the CHIT/G–CN microsensor exhibited the highest sensitivity, with a detection limit of 1.22 × 10^−7^ mol/L. This high sensitivity makes it particularly suitable for detecting quercetin in complex botanical matrices, such as Ginkgo biloba oil, where active compound concentrations may vary. The G–CN and Co(II)TPP/G–CN sensors also performed well, with detection limits of 5.64 × 10^−8^ mol/L and 9.80 × 10^−8^ mol/L, respectively. These detection limits are within the range required for practical applications in cosmeceutical formulations, where even trace amounts of quercetin can have significant biological effects.

Achieving low detection limits is critical for ensuring the efficacy of cosmeceutical products. Quercetin, when applied topically, must penetrate the skin barrier and reach the deeper layers of the epidermis to exert its antioxidant and anti-inflammatory effects. Reliable methods for measuring its concentration in formulations are essential for product development and quality control. The recovery rates and relative standard deviation (RSD) values obtained in this study (97.4% ± 0.43) further demonstrate the accuracy and precision of the microsensors, providing confidence in their real-world applicability.

While our approach offers promising results, it is important to consider the advantages of electrochemical detection in comparison with other established methods for quercetin analysis. High-performance liquid chromatography (HPLC), mass spectrometry, and spectrophotometry are all widely used for the quantification of quercetin. HPLC is known for its high accuracy and ability to separate quercetin from complex matrices, but it requires expensive equipment and skilled personnel. Mass spectrometry provides detailed structural information but is similarly costly and time-consuming. Spectrophotometry offers a simpler, faster alternative, but its sensitivity is often lower than that of electrochemical methods. In contrast, electrochemical detection provides a sensitive, cost-effective, and portable solution for quercetin analysis. The use of DPV in this study offers superior sensitivity, particularly at low concentrations, and the microsensors developed here are more affordable and accessible compared to traditional analytical techniques.

Despite its advantages, electrochemical detection does have limitations. The sensitivity of the microsensors can be affected by matrix interferences in real-world samples, and the stability of the sensors over time may need to be improved. Furthermore, the performance of these sensors could be enhanced by optimizing the materials used in sensor construction or exploring alternative modifications. Future research could focus on improving sensor stability and exploring additional modifications to increase selectivity and reduce the influence of matrix interferences.

The findings of this study also open the door to further research into the use of quercetin and other flavonoids in regenerative medicine and therapeutic treatments. As the interest in natural, plant-based compounds grows, innovative methods for harnessing their potential are essential. The combination of advanced electrochemical techniques with sustainable sourcing, as demonstrated in this study, offers a promising avenue for developing cosmeceuticals that are not only effective, but also environmentally responsible. Quercetin has already shown regenerative properties in skin cell renewal and wound healing [35], and the use of electrochemical techniques may further enhance our understanding of its role in these processes. Additionally, the research by Sasounian et al. (2023) emphasizes the importance of combining natural compounds with sustainable practices, advocating for eco-friendly and effective skincare solutions [36].

In conclusion, this study contributes to the growing body of research on natural antioxidants in cosmeceuticals, particularly focusing on quercetin’s electrochemical behavior in *Ginkgo biloba* essential oil. The development of highly sensitive microsensors provides valuable tools for detecting and quantifying quercetin in botanical extracts, enabling its integration into next-generation skincare products. By combining the therapeutic potential of plant-derived compounds with advanced electrochemical analysis, we can create innovative solutions that promote both human health and environmental sustainability.

The novelty of this work lies in the development of innovative amperometric microsensors for the precise detection of quercetin in Ginkgo biloba essential oil, a complex botanical matrix. Key aspects of the novelty include:

Design of new microsensors: this study introduces three novel amperometric microsensors based on graphite (G) and carbon nanoparticles (CNs), with modifications using chitosan (CHIT) and cobalt(II) tetraphenylporphyrin (Co(II)TPP). These modifications enhance the sensors’ sensitivity, selectivity, and electrocatalytic activity, making them highly effective for detecting quercetin at low concentrations.

Use of differential pulse voltammetry (DPV): this study applies the DPV technique to quercetin detection in a botanical matrix, demonstrating the high resolution and sensitivity of this electrochemical method, which is well-suited for trace-level analysis in complex samples, like plant extracts. This represents a novel application of DPV for quercetin in cosmeceutical and pharmaceutical contexts.

Enhanced detection limits: this study achieves exceptionally low detection limits (down to 1.22 × 10^−7^ mol/L for the CHIT/G-CN sensor), which is important for accurately measuring quercetin in cosmeceutical formulations, where even trace amounts of quercetin can have significant biological effects.

Exploration of electrochemical sensors for flavonoids in botanical extracts: while electrochemical methods for flavonoids are not new, this work specifically targets quercetin, a prominent antioxidant, in *Ginkgo biloba* oil, providing insights into its electrochemical behavior and offering a reliable tool for monitoring its bioavailability and effectiveness in skincare applications.

Potential for sustainable, eco-friendly cosmeceuticals: by combining cutting-edge electrochemical techniques with sustainable sourcing practices (like using plant-derived compounds), this work addresses the growing demand for natural, eco-friendly skincare solutions, paving the way for the development of regenerative medicines and therapeutic treatments based on plant antioxidants like quercetin.

The detection of quercetin in cosmetics can play a significant role in the development of metabolite science for several reasons, despite quercetin typically being used in unchanged form in cosmetic products. Here’s how the study of quercetin’s presence, quantification, and behavior in cosmetics can contribute to metabolite science:

1. Understanding quercetin’s bioavailability and metabolism

While quercetin in cosmetics is applied in its unchanged form, it is well-documented that, once absorbed through the skin, quercetin undergoes metabolic transformations. These transformations are crucial for its therapeutic effects, as the metabolites of quercetin often exhibit different or enhanced biological activities compared to the parent compound. For example, quercetin is metabolized into various conjugates (e.g., glucuronides, sulfates, and methylated forms) within the skin and other tissues, which can affect its antioxidant, anti-inflammatory, and cardioprotective properties.

By developing accurate methods to measure quercetin in cosmetics, this study opens the door to monitoring its metabolism and bioavailability after topical application. Knowing how quercetin behaves in the skin, including how it is metabolized into active forms, provides essential information for optimizing the efficacy of quercetin-based cosmeceuticals and advancing personalized skincare. This is critical in metabolite science, as understanding the biotransformation of quercetin within skin cells (and other organs) leads to improved formulations and better therapeutic outcomes.

2. Linking cosmetic applications with systemic effects

The metabolites of quercetin have shown activity in other organs beyond the skin. For example, quercetin metabolites are involved in regulating blood pressure, modulating vascular function, and providing anti-inflammatory effects. By detecting quercetin in cosmetic formulations, researchers can track its percutaneous absorption and systemic distribution, enabling a better understanding of how topical quercetin may influence the body’s metabolism beyond just the skin.

3. Improved development of topical therapies

Accurate detection of quercetin in cosmetic products could enhance the development of topical therapies that are not just aimed at skin benefits, but also take into account the systemic effects of quercetin metabolites. If quercetin’s metabolites are found to significantly impact other organs, such as the heart or liver, this could open up new directions for cosmeceutical formulations that provide multi-organ benefits, which is a growing trend in the skincare and pharmaceutical industries.

4. Link between cosmetics and metabolite profiling

While quercetin itself is applied in unchanged form, studying its metabolite profiles after skin absorption could lead to better formulations and formulations that actively contribute to skin health and systemic wellbeing. With improved detection methods for quercetin in cosmetics, researchers can build detailed profiles of its metabolites, contributing to the advancement of metabolite science by linking topical application to systemic metabolic effects.

5. Potential for personalized skin care

The development of detection systems for quercetin in cosmetics can ultimately help scientists better understand individual variations in metabolism—how different people metabolize quercetin and how this may affect the efficacy of topical treatments. This could eventually contribute to personalized skincare solutions, where consumers are provided with products tailored to their specific metabolic profiles, improving their overall health and skincare results.

While quercetin is used in its unchanged form in cosmetics, studying its metabolism and detection through improved electrochemical sensors will significantly contribute to metabolite science. This research will allow a deeper understanding of how quercetin and its metabolites interact with the skin and other organs, contributing to more effective topical therapies, personalized skincare, and the advancement of metabolomics. The ability to measure quercetin and its metabolites in complex matrices, like cosmetics, is a key step toward integrating metabolite science with cosmetic product development and therapeutic applications.

## 5. Conclusions

The increasing integration of plant-based ingredients in pharmaceutical and cosmeceutical products is a response to both consumer demand for natural solutions and the growing recognition of the efficacy of plant-derived compounds in addressing a wide range of health concerns. Quercetin, a flavonoid with well-documented antioxidant, anti-inflammatory, and antimicrobial properties, has emerged as a key bioactive molecule with applications in skincare and internal health management. Our research aimed to further understand its behavior in a novel botanical medium—*Ginkgo biloba* essential oil—harvested from a regenerative farming system. The emphasis on regenerative farming not only ensures the purity of the plant material, but also aligns with the principles of environmental sustainability, which is increasingly important in both consumer preferences and the scientific research.

The unique value of *Ginkgo biloba* essential oil lies in its composition of bioactive compounds that synergize with quercetin, enhancing its therapeutic potential. As such, our study focused on the electrochemical behavior of quercetin within this specific matrix, which has not been previously investigated. This approach allowed us to explore how quercetin behaves in a complex natural extract and assess its potential applications in the cosmetics industry, particularly for products designed to treat skin disorders, allergies, and oxidative stress-related conditions.

One of the key challenges in harnessing the potential of quercetin in cosmeceutical formulations is ensuring its stability, bioavailability, and effectiveness when applied topically. While quercetin is known to exhibit potent biological activity, its integration into skincare products requires that it be efficiently absorbed into the skin. Electrochemical studies, such as ours, are essential for optimizing detection methods that can reliably measure the concentration of quercetin in these formulations, ensuring that the active ingredient is present in sufficient quantities to deliver the desired therapeutic effects.

## Figures and Tables

**Table 1:** Response characteristics of the amperometric microsensors used for quercetin (Q) analysis.

Amperometric Microsensor	Equation for Calibration	Sensitivity (A µmol L^−1^)	Limit of Detection (µmol L^−1^)	Linear Concentration Range (µmol L^−1^)
G-CN	H = 5.64 × 10^−8^ + 1.44 × 10^−6^ × C r = 0.99584	5.64 × 10^−8^	0.0001	0.0001–0.01
CHIT/G-CN	H = 1.22 × 10^−7^ + 4.61 × 10^−7^ × C r = 0.99475	1.22 × 10^−7^	0.001	0.001–1
CoP/G-CN	H = 9.80 × 10^−8^ + 3.34 × 10^−7^ × C r = 0.99854	9.80 × 10^−8^	0.0001	0.0001–1

**Table 2:** Recovery rates of quercetin (Q) in Gingko biloba essential oil of cosmetic interest.

Amperometric Microsensor	Recovery (±RSD) (%)
G-CN	97.4 ± 0.43
CHIT/G-CN	91.8 ± 0.56
CoP/G-CN	90.5 ± 0.36

## Data Availability

The original contributions presented in the study are included in the article. Further inquiries can be directed to the corresponding authors.
